# MGST1 drives lymph node metastasis in papillary thyroid carcinoma via mitochondrial metabolic reprogramming and immune suppression

**DOI:** 10.3389/fimmu.2026.1848083

**Published:** 2026-06-04

**Authors:** Qing-Xuan Wang, Chang-Feng Yu, Ke-Chen Zhang, Jun-Hao Zhang, Xu-Jie Zhou, Tong-Xin Zou, Hao-Zhuo Huang, Tian-Yu Han, Hua-Zhen Mei, Kai-Bo Su, Jie You, Jin-Miao Qu, Cong Xie, Quan Li, Ye-Feng Cai

**Affiliations:** 1 Department of Thyroid Surgery, The First Affiliated Hospital of Wenzhou Medical University, Wenzhou, Zhejiang, China; 2 Zhejiang Key Laboratory of Intelligent Cancer Biomarker Discovery and Translation, First Affiliated Hospital, Wenzhou Medical University, Wenzhou, China; 3 School of Ophthalmology & Optometry, Wenzhou Medical University, Wenzhou, Zhejiang, China; 4 The First Clinical Medical College, Wenzhou Medical University, Wenzhou, Zhejiang, China; 5 Department of General Surgery, Zhejiang Dinghai Hospital, Shanghai Ruijin Hospital Zhoushan Branch, Zhoushan, Zhejiang, China; 6 Department of Breast Surgery, The First Affiliated Hospital of Wenzhou Medical University, Wenzhou, Zhejiang, China

**Keywords:** immune evasion, lymph node metastasis, metabolic reprogramming, MGST1, papillary thyroid carcinoma

## Abstract

**Background:**

While papillary thyroid carcinoma (PTC) generally exhibits a favorable prognosis, a subset of patients experiences poorer outcomes, with lymph node metastasis (LNM) significantly increasing the risk of recurrence and correlating with a worse prognosis. Mitochondrial metabolic reprogramming and immune evasion play pivotal roles in driving lymph node metastasis; however, the specific actionable targets within these processes remain largely unexplored.

**Methods:**

We constructed a multi-omics discovery framework by integrating TCGA/GTEx transcriptomics, a large-scale independent clinical cohort, and single-cell RNA sequencing. Both mitochondrial metabolic signatures and immune evasion landscapes were investigated to characterize their roles in driving LNM. A consensus machine learning framework was deployed to isolate core drivers, which were rigorously validated through genetic manipulation and pharmacological assays.

**Results:**

Unsupervised clustering identified a high-risk “Mito-high” subtype, characterized by distinct metabolic reprogramming and an immunosuppressive microenvironment, in which CD8^+^ T cell depletion coincided with Treg enrichment. Machine learning prioritized MGST1 as the core predictor, consistently ranking as a top feature in both the integrated TCGA/GTEx dataset and our independent cohort. The MGST1-based model demonstrated robust predictive performance, achieving an AUC of 0.833 in external validation. At the single-cell level, trajectory analysis positioned MGST1 at the terminal stage of dedifferentiation, marking a stem-like metastatic subpopulation. Pharmacological inhibition of MGST1 with Toxoflavin dose-dependently suppressed proliferation, migration, and invasion while inducing apoptosis. Critically, pharmacological inhibition of MGST1 may reverse the “immune-cold” phenotype by promoting IL-1β release and suppressing TGF-β1. siRNA-mediated knockdown confirmed the target dependency of Toxoflavin.

**Conclusion:**

Our findings uncover a previously unrecognized link between mitochondrial metabolic reprogramming, immune evasion and LNM in PTC and establish MGST1 as a robust biomarker for metastatic risk stratification and a pivotal metabolic-immune regulator. Moreover, the target-specific activity of toxoflavin provides a rational therapeutic strategy for patients with high-risk PTC characterized by MGST1-driven mitochondrial reprogramming and immune evasion.

## 1 Introduction

Papillary thyroid carcinoma (PTC) is the most prevalent subtype of thyroid cancer, accounting for approximately 85% of all thyroid malignancies (1–3). Lymph node metastasis (LNM) is frequently observed at the time of diagnosis and is associated with an increased risk of recurrence, therapeutic resistance, and overall poor outcomes (4–6). About 40% to 60% patients with PTC present with LNM at diagnosis (7–9). Therefore, identifying molecular determinants of PTC’s lymph node metastatic potential is critical for improving risk stratification and guiding personalized treatment.

Emerging evidence suggests that metabolic reprogramming and immune evasion are hallmarks of metastasis, enabling tumor cells to survive when detached and colonize distant sites (10–12). Mitochondria play a pivotal role in these processes. Far from being simple bioenergetic “powerhouses,” mitochondria are now recognized as pivotal coordinators of cancer progression and immune landscape remodeling (13–17). For instance, osteocytes transfer mitochondria to metastatic cancer cells and trigger the cGAS/STING-mediated antitumor immune response. Blocking mitochondrial transfer by specifically knocking out mitochondrial Rho GTPase 1 (Rhot1) or mitochondrial mitofusin 2 (Mfn2) in osteocytes can impair tumor immunogenicity (18). Furthermore, alterations in mitochondrial DNA copy number and somatic mutations have been associated with the initiation, progression, and metastasis of various cancer types (19–21). However, the specific mechanisms by which mitochondria-related genes (MRGs) supports the lymphatic dissemination of PTC remain poorly understood, underscoring the need to explore their roles in PTC.

In this study, driven by our initial observation that LNM-associated genes are enriched in mitochondrial pathways, we integrated multi-omics data to identify key mitochondrial drivers systematically. Among these, microsomal glutathione S-transferase 1 (MGST1) emerged as a critical gene in PTC progression, highlighting its potential as a diagnostic marker and therapeutic target.

## 2 Materials and methods

### 2.1 Data sources and processing

To establish a platform-independent transcriptomic baseline, we integrated 792 samples (459 PTC and 333 normal) from TCGA and GTEx via the UCSC Xena platform. Raw data were harmonized using the Toil pipeline and standardized to log_2_(TPM + 1), with only the highest-expressing isoforms retained for downstream analysis.

For independent external validation, a cohort comprising 280 PTC tissue samples paired with long-term follow-up data was retrospectively curated from the First Affiliated Hospital of Wenzhou Medical University. Within this population, a high-quality subset of 155 patients possessing comprehensive clinicopathological annotations, specifically BRAF mutation status, extrathyroidal extension (ETE), and histological subtypes, was stratified for multivariate regression analyses to ensure robust statistical power. The study protocol adhered to ethical guidelines (Approval No. KY2024-004), and written informed consent was obtained from all participants.

### 2.2 Bulk RNA-seq and functional enrichment analysis

Differentially expressed genes (DEGs) were identified using the limma R package (adjusted *p* < 0.05, |log_2_FC| > 1). Functional annotations, including Gene Ontology (GO) and Kyoto Encyclopedia of Genes and Genomes (KEGG) analyses, were performed via the clusterProfiler package. To characterize metabolic heterogeneity, we conducted Gene Set Variation Analysis (GSVA) and Single-sample Gene Set Enrichment Analysis (ssGSEA) based on established metabolic signatures. Subsequently, Non-negative Matrix Factorization (NMF) clustering was applied to the mitochondria-related DEGs (rank = 2–7, nrun = 500) to stratify patients into molecular subtypes.

### 2.3 Immune profiling and gene correlation

The immune landscape was deconvoluted using CIBERSORT (LM22 signature, 1,000 permutations) and xCell algorithms, complemented by ssGSEA for quantifying immune cell infiltration and pathway activities across different MGST1 expression groups. Interactions among mitochondrial scores, metabolic signatures, and immune infiltration profiles were subsequently evaluated via Spearman correlation analysis.

### 2.4 Machine learning framework

A machine learning workflow was orchestrated via the caret package to screen LNM predictors, commencing with feature reduction using Least Absolute Shrinkage and Selection Operator (LASSO) regression (glmnet). Ten distinct algorithms—including Generalized Linear Model (GLM), Neural Network (NNET), Random Forest (RF), Gradient Boosting Machine (GBM), Support Vector Machine (SVM), eXtreme Gradient Boosting (XGB), k-Nearest Neighbors (KNN), AdaBoost (ADA), Classification and Regression Trees (CART), and Multilayer Perceptron (MLP)—were subsequently trained and rigorously validated across the internal TCGA dataset and the independent in-house cohort (n=280).

### 2.5 Weighted gene co-expression network analysis

Gene co-expression networks were constructed using the WGCNA R package to isolate modules significantly associated with clinical traits. A soft-thresholding power was determined to achieve a scale-free topology fit index of 0.9. Subsequently, modules showing significant correlation with LNM status were identified, and the module membership of candidate genes was interrogated within the relevant networks.

### 2.6 Single-cell RNA-seq analysis

Single-cell transcriptomes from 22 PTC samples (GSE184362) were processed via the Seurat pipeline, applying stringent exclusion criteria for low-quality cells (<300 genes, <1,000 UMIs, or >20% mitochondrial reads). Following batch-effect removal with Harmony, dimensionality reduction was performed using PCA and t-SNE/UMAP. Evolutionary trajectories and differentiation states were reconstructed using Monocle (DDRTree method) and CytoTRACE, while branch-specific transcriptional changes were interrogated via Branched Expression Analysis Modeling (BEAM). Furthermore, intercellular communication networks were inferred using CellChat (v2.1.2) and CellPhoneDB (v5.0.0) algorithms. CellChat was utilized to identify macroscopic signaling pathway alterations, while CellPhoneDB was applied to map specific receptor-ligand interactions between tumor subpopulations and immune cells based on established databases.

### 2.7 Cell culture and drug treatment

Human PTC cell lines (KTC1 (BRAF V600E mutant type) and TPC1 (BRAF V600E wild type)), procured from the Stem Cell Bank, Chinese Academy of Sciences, were maintained in RPMI-1640 medium supplemented with 10% fetal bovine serum (FBS) under standard conditions (37 °C, 5% CO_2_). For pharmacological interventions, Toxoflavin (TargetMol, USA) was reconstituted in DMSO and administered at indicated concentrations, alongside vehicle-matched controls.

### 2.8 Cell transfection

Genetic silencing was achieved by transfecting cells with MGST1-specific siRNAs (Si-1/2) or negative controls (Tsingke Biotech Co., Ltd. Beijing, China) using Lipofectamine 3000 (Invitrogen, USA). Knockdown efficiency was confirmed via Western blot and qRT-PCR at 48 h; for subsequent drug sensitivity assays, Toxoflavin treatment was initiated 24 h post-transfection.

### 2.9 RNA extraction and quantitative real-time PCR

Total RNA was isolated from biological specimens—including frozen tissues and cultured cell lines—using TRIzol reagent (Invitrogen, USA), immediately followed by complementary DNA (cDNA) synthesis via the PrimeScript RT Reagent Kit (Takara, Japan). Quantitative amplification was subsequently monitored on a LightCycler 480 system (Roche) employing SYBR Green chemistry. Relative MGST1 transcript abundance was determined using the 2^-ΔΔCt^ method, normalized to the internal control *GAPDH*. Primer sequences were as follows: MGST1: 5′- TCCTGCACTTCAGACTATTTGT -3′(forward) and 5′- CCTGTAAGCCATGGAAAGAGTA -3′(reverse); GAPDH: 5′-GGTCGGAGTCAACGGATTTG-3′(forward) and 5′-ATGAGCCCCAGCCTTCTCCAT -3′(reverse).

### 2.10 Western blot

Protein lysates were harvested using RIPA buffer supplemented with protease inhibitors. Following electrophoretic separation via SDS-PAGE, proteins were blotted onto PVDF membranes and probed overnight at 4 °C with primary antibodies against MGST1 (HM0311, HUABIO) and GAPDH (60004-1-Ig, Proteintech). Immunoreactive bands were subsequently visualized using an ECL detection system after incubation with HRP-conjugated secondary antibodies.

### 2.11 Cell viability and drug sensitivity assays

Cell proliferation kinetics were monitored using the Cell Counting Kit-8 (CCK-8; Beyotime, Shanghai, China) by measuring absorbance at 450 nm over a 96-hour interval. For pharmacological profiling, Toxoflavin (T17142, TargetMol) was reconstituted in DMSO. To determine target-dependent sensitivity, cells were pre-transfected with si-NC or si-MGST1 for 24 h, re-seeded, and subsequently challenged with graded concentrations of Toxoflavin for an additional 48 h. Half-maximal inhibitory concentrations (IC_50_) were derived via non-linear regression (GraphPad Prism 8.0), while phenotypic rescue was quantified by calculating cell death rates (1 - Cell Viability) ×100% at indicated dosages.

### 2.12 Colony formation

Cells were plated at low density in 6-well dishes. Following a 10–14-day incubation period, the resulting colonies were fixed with 4% paraformaldehyde and stained with 0.1% crystal violet.

### 2.13 Migration and invasion

Migration and invasion potentials were evaluated using Transwell chambers (Corning, USA) with Matrigel-coated inserts for invasion assays. Cells suspended in serum-free medium were plated in the upper inserts, while the lower reservoir was filled with medium containing 10% FBS serving as a chemoattractant. Following an incubation period of 24 hours for the migration assay and 48 hours for the invasion assay, the transferred cells were fixed with 4% paraformaldehyde and stained with 0.1% crystal violet.

### 2.14 Flow cytometry analysis of apoptosis

To assess drug-induced apoptosis in the context of MGST1 depletion, transfected cells were treated with Toxoflavin for 48 h. Cells were subsequently harvested and dual-stained with Annexin V-FITC and Propidium Iodide (BD Biosciences) for 15 min in the dark, followed by flow cytometric quantification on a Beckman Coulter system.

### 2.15 Quantification of extracellular IL-1β and TGF-β1 levels

Supernatant IL-1β levels were determined by ELISA (MSKBIO Technology, China) with absorbance read at 450 nm, while TGF-β1 protein levels were detected using an Human TGF-β1 ELISA Kit (PT880, Beyotime Biotechnology, China) following the recommended protocol, also with absorbance measurement at 450 nm.

### 2.16 Statistical analysis

Data were analyzed using R (v4.3.3) and GraphPad Prism 8.0, and quantitative results are presented as mean ± standard deviation (SD) from at least 3 independent biological replicates. Two-group comparisons employed Student’s t-test or the Wilcoxon rank-sum test, whereas multi-group differences were evaluated via one-way or two-way ANOVA followed by Tukey’s *post hoc* correction. IC50 values were modeled using non-linear regression, with significance thresholds set at *p* < 0.05.

## 3 Results

### 3.1 Mitochondrial metabolic reprogramming drives lymph node metastasis and defines a high-risk prognostic subtype

**Figure 1:**
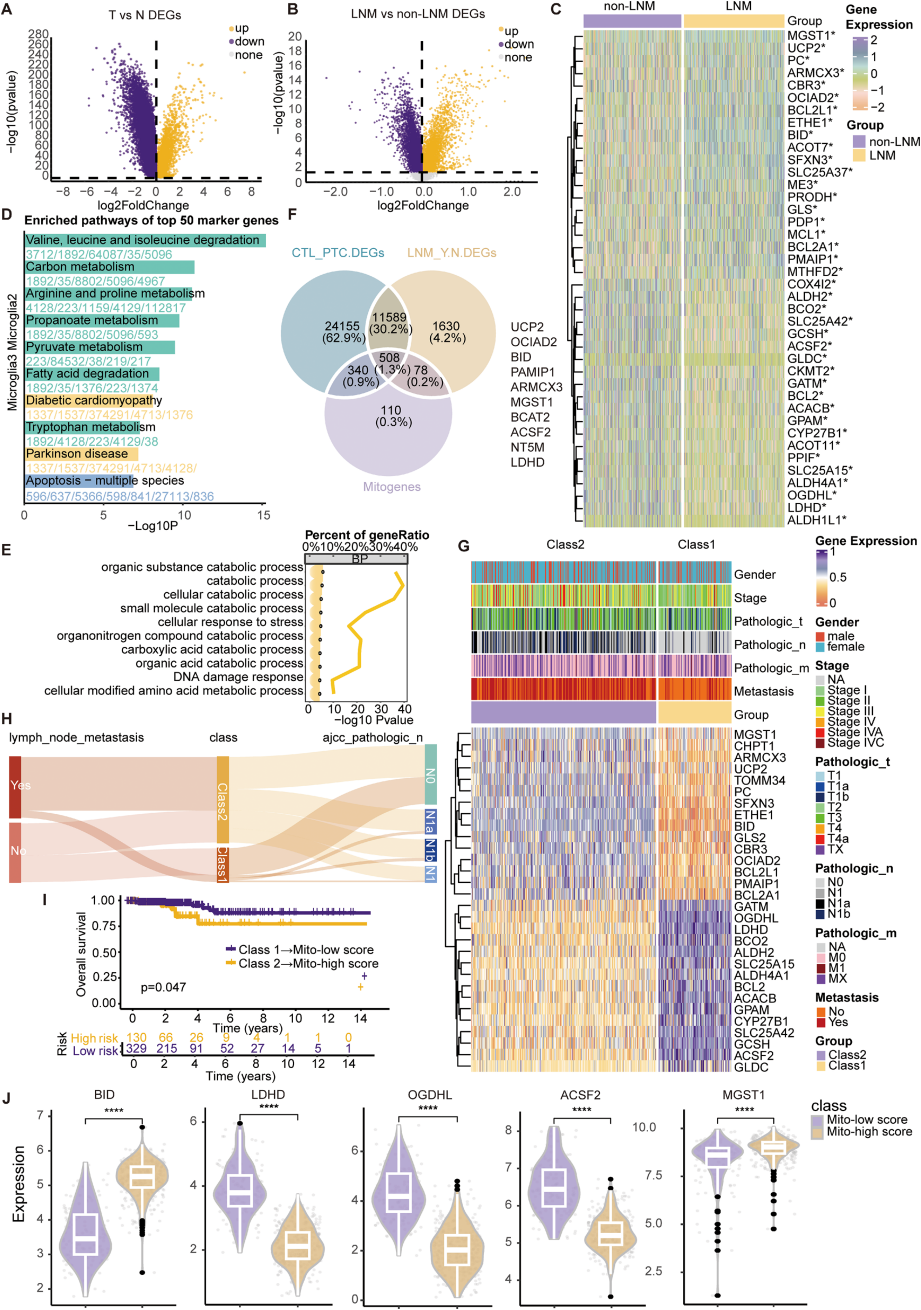
Transcriptomic landscape reveals mitochondria-driven metabolic reprogramming and establishes a prognostic subtype in PTC. **(A, B)** Volcano plots illustrating differentially expressed genes (DEGs) between tumor and normal tissues **(A)** and between LNM-positive and LNM-negative samples **(B)**. **(C)** Hierarchical clustering heatmap showing the distinct transcriptomic separation of LNM-positive samples based on top DEGs. **(D)** Bar plot displaying the top enriched pathways of marker genes, highlighting mitochondrial metabolic processes. **(E)** Gene Ontology (GO) enrichment analysis showing the top enriched biological processes related to catabolism. **(F)** Venn diagram identifying 508 overlapping mitochondria-related genes (MRGs) by intersecting tumor-specific DEGs, LNM-associated DEGs, and the mitochondrial gene set. **(G)** Heatmap of NMF consensus clustering based on the 508 MRGs, stratifying the cohort into Class 1 (Mito-low) and Class 2 (Mito-high) subtypes with corresponding clinicopathological features (top annotations). **(H)** A Sankey diagram illustrates the distribution of lymph node metastasis status and AJCC pathologic stage between the two molecular subtypes. **(I)** Kaplan-Meier curve comparing overall survival (OS) between Class 1 and Class 2 subtypes (*p* = 0.047). **(J)** Violin plots comparing the expression levels of representative mitochondrial drivers (e.g., BID, LDHD, OGDHL, ACSF2, MGST1) between the two subtypes. ^****^P < 0.0001.

To ensure the robustness of our transcriptomic landscape, we first performed rigorous data preprocessing and batch correction across datasets (**Supplementary Figures 1A–C**). Based on this harmonized data, transcriptomic profiling initially confirmed widespread molecular alterations between tumor and normal tissues (**Figure 1A**). More critically, beyond this tumorigenic baseline, we identified a distinct divergent expression profile distinguishing LNM-positive from LNM-negative tumors (**Figures 1B, C**), thereby delineating a specific signature driving metastasis. Subsequent functional enrichment analyses consistently pinpointed mitochondrial dysfunction, characterized by specific alterations in valine/leucine degradation and fatty acid metabolism, as the core metabolic engine of this aggressive phenotype (**Figures 1D, E**).

Intersection of LNM-associated signatures with the mitochondrial gene set identified 508 metastasis-specific mitochondria-related genes (MRGs) (**Figure 1F**). Leveraging these drivers, we employed unsupervised NMF clustering to stratify the cohort. Guided by comprehensive stability metrics, including cophenetic and silhouette scores, we determined the optimal cluster number as k=2 (**Supplementary Figures 1D–F**), a classification further validated by the distinct spatial separation observed in t-SNE analysis (**Supplementary Figure 1G**). Unsupervised NMF clustering based on these MRGs stratified the cohort into two robust molecular subtypes, with Class 2 exhibiting aberrant high expression of these drivers (**Figure 1G**). Clinical analysis confirmed that Class 2 effectively captured patients with aggressive features: this subgroup bore a significantly higher burden of LNM and advanced pathologic stages (**Figure 1H**). Crucially, Class 2 was significantly associated with poorer overall survival (p=0.047, **Figure 1I**). Accordingly, we designated Class 2 as the high-risk “Mito-high” subtype. The significant upregulation of key drivers such as MGST1 and UCP2 in this group (**Figure 1J**) further validated the precision of our subtyping system in resolving metabolic heterogeneity and exposing a potential therapeutic vulnerability.

### 3.2 Metabolic reprogramming and immune evasion characterize the high-risk “Mito-high” subtype

**Figure 2:**
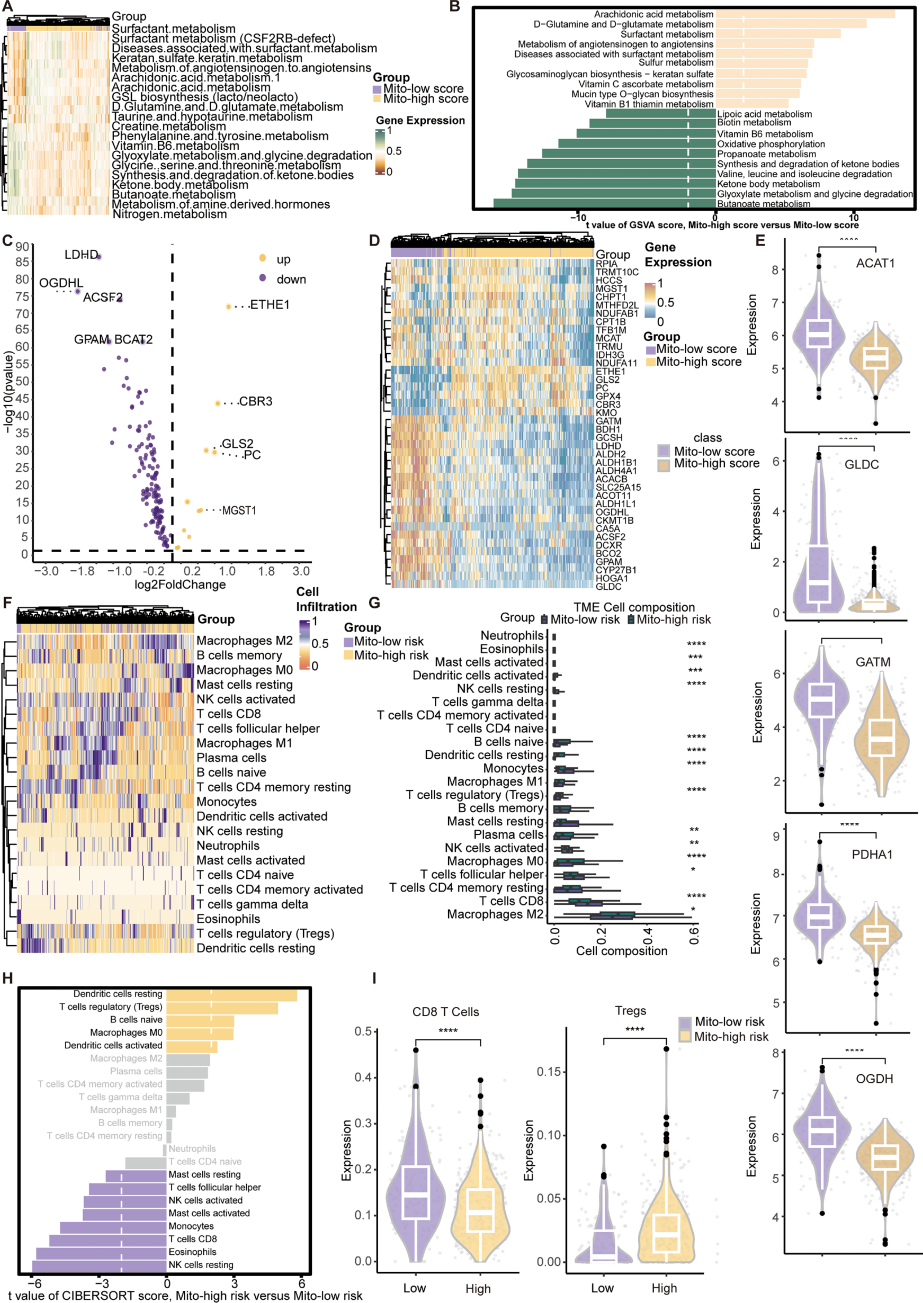
Metabolic reprogramming and immune evasion characterize the high-risk “Mito-high” subtype. **(A, B)** GSVA enrichment analysis **(A)** and bar plot **(B)** illustrating profound and widespread metabolic reprogramming in the high-risk subtype (Class 2) compared to the low-risk group (Class 1). **(C)** Volcano plot of differentially expressed metabolism-related genes between the two subtypes. **(D)** Heatmap visualizing the distinct expression patterns of metabolic regulators. **(E)** Violin plots quantifying the expression of representative metabolic genes, confirming the subtype-specific metabolic stratification. **(F)** Heatmap of the immune landscape depicting the differential infiltration of immune cells. **(G, H)** CIBERSORT analysis displaying the composition of infiltrating immune cells **(G)** and the difference in cell abundance **(H)** between subtypes. **(I)** Violin plots confirm an immunosuppressive microenvironment in the high-risk subtype, characterized by CD8+ T-cell depletion and regulatory T-cell (Treg) enrichment. ^****^P < 0.0001.

GSVA analysis unveiled a profound and widespread metabolic reprogramming in the Mito-high subtype (Class 2), establishing this systemic metabolic shift as the intrinsic basis of its aggressive phenotype (**Figures 2A, B**). In sharp contrast to the Mito-low subtype, which maintained active oxidative phosphorylation, the Mito-high subtype was distinguished by the aberrant activation of tumor-promoting metabolic pathways. This pathway-level alteration was substantiated by a panoramic dysregulation of metabolic regulators, as visualized in the volcano plot and heatmap (**Figures 2C, D**). Quantitative analysis of representative metabolic genes further confirmed this subtype-specific stratification (**Figure 2E**), indicating that the high-risk phenotype relies on a distinct metabolic architecture to fuel its progression.

Investigating the immune microenvironment revealed that this metabolic rewiring was coupled with extensive immune remodeling (**Figure 2F**). Decomposition of immune infiltrates identified a striking “cold” tumor phenotype in the Mito-high subtype. Unlike the Mito-low group, which was enriched with anti-tumor effectors including CD8^+^ T cells and activated NK cells (**Figures 2G, H**), the Mito-high subtype exhibited a marked depletion of cytotoxic CD8^+^ T cells with a concurrent enrichment of immunosuppressive regulatory T cells (Tregs) (**Figure 2I**). Collectively, these findings define the high-risk mitochondrial subtype by a specific “double-hit” mechanism: systemic metabolic reprogramming coupled with an immunosuppressive microenvironment, thereby fostering tumor escape and metastasis.

### 3.3 Machine learning prioritizes MGST1 as a robust, independent predictor of LNM and aggressive tumor burden

**Figure 3:**
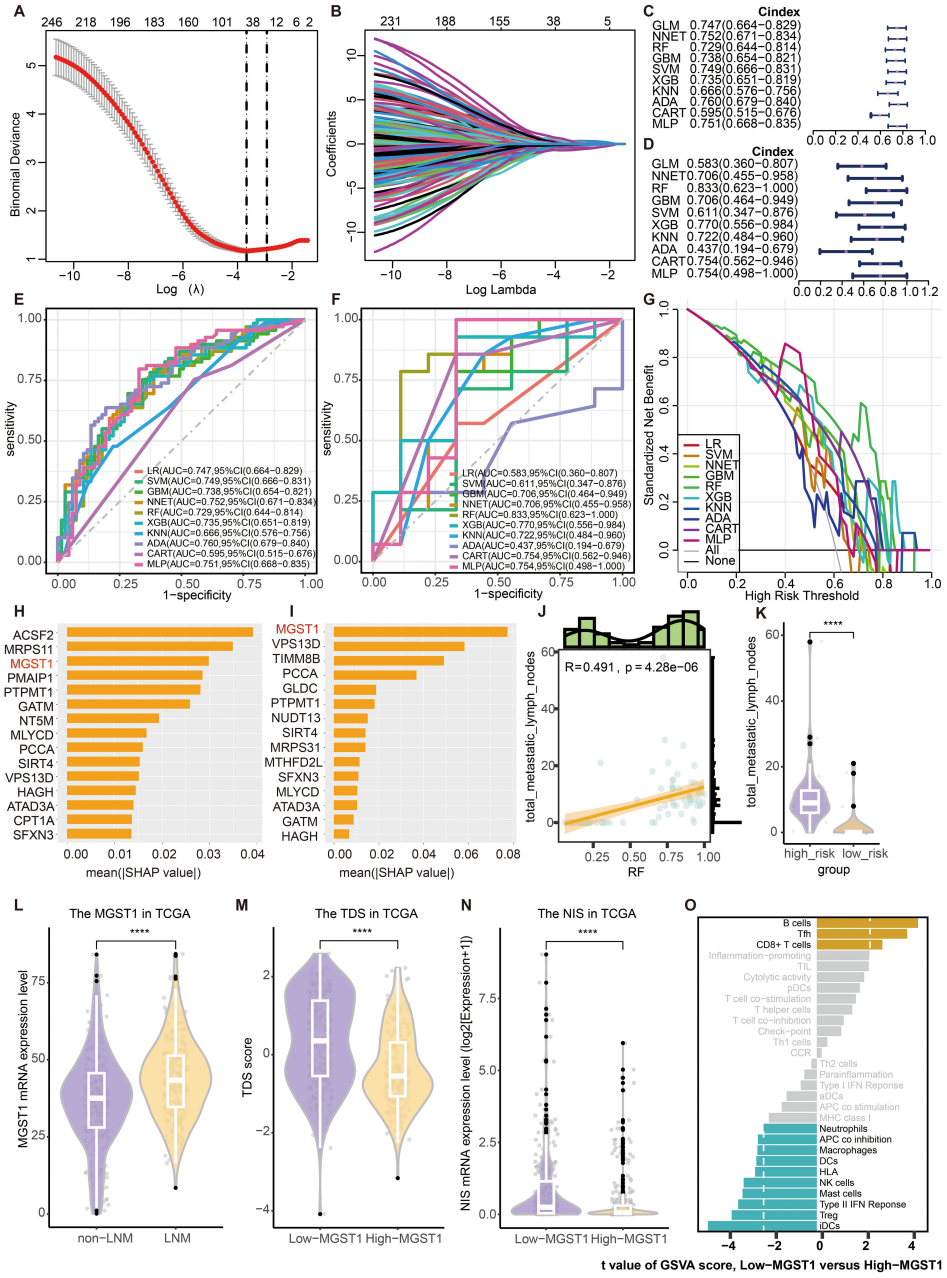
Machine learning prioritizes MGST1 as a robust, independent predictor of LNM and aggressive tumor burden. **(A, B)** LASSO regression analysis illustrating the selection of the optimal tuning parameter (λ) **(A)** and coefficient profiles of candidate features **(B)**. **(C, D)** Forest plots comparing the Concordance Index (C-index) of ten machine learning algorithms in the TCGA training cohort **(C)** and the independent in-house validation cohort **(D)**, identifying Random Forest (RF) as the superior model. **(E, F)** Receiver Operating Characteristic (ROC) curves demonstrating the robust diagnostic performance of the RF model in the TCGA **(E)** and external validation cohorts **(F)**. **(G)** Decision Curve Analysis (DCA) evaluates the clinical net benefit of the RF model across different threshold probabilities. **(H, I)** SHAP summary plots ranking feature importance, consistently prioritizing MGST1 as a top contributor to LNM prediction in both TCGA **(H)** and in-house **(I)** cohorts. **(J)** Scatter plot showing the positive correlation between the RF-derived risk score and the total metastatic lymph node count (R = 0.491, *p* = 4.28×10^-6^). **(K)** Violin plot confirms that patients in the high-risk group bear a significantly higher metastatic burden. **(L)** Violin plot validating the significant upregulation of MGST1 mRNA in LNM-positive tissues compared to non-LNM tissues. **(M, N)** Violin plots illustrating the negative association of MGST1 high expression with Thyroid Differentiation Score (TDS) **(M)** and SLC5A5 (NIS) expression **(N)**. **(O)** Bar plot illustrates the differential immune cell infiltration (e.g., CD8+ T cells and Tregs) between the low- and high-MGST1 groups evaluated by ssGSEA. ^****^P < 0.0001.

To construct a clinically applicable prediction tool, we implemented an ensemble machine learning framework incorporating LASSO-based feature selection (**Figures 3A, B**). A comprehensive head-to-head comparison of 10 algorithms identified the Random Forest (RF) model as the superior predictor. C-index assessment demonstrated that the RF model consistently exhibited optimal prognostic accuracy across both the TCGA cohort (**Figure 3C**) and our independent in-house validation cohort (n=280) (**Figure 3D**), underscoring its robustness in capturing LNM heterogeneity. This predictive power was substantiated by ROC analysis, where the model achieved a high AUC in the TCGA cohort (**Figure 3E**) and, notably, maintained excellent stability in the external validation setting (**Figure 3F**). Furthermore, Decision Curve Analysis (DCA) indicated that the RF model provided a significant net clinical benefit over broad intervention strategies (**Figure 3G**). SHAP analysis revealed the decision-making logic of the “black box” model, consistently prioritizing MGST1 as the top contributing feature in both cohorts, with higher expression levels driving higher risk prediction (**Figures 3H, I**). This finding was independently corroborated by WGCNA, which mapped MGST1 to the gene module most strongly correlated with LNM (**Supplementary Figure 2**). Beyond binary prediction, the RF risk score correlated positively with the total number of metastatic lymph nodes (**Figure 3J**), confirming that patients in the high-risk group bore a significantly higher metastatic burden (**Figure 3K**).

Clinical investigation finally established MGST1 as a specific determinant of LNM. MGST1 mRNA expression was significantly upregulated in LNM-positive tissues compared to non-LNM tissues (**Figure 3L**). We analyzed clinicopathological features in both the TCGA (n=499) and our independent, annotated cohort (n=155). In the TCGA cohort, high MGST1 expression was strongly associated with aggressive features, including lymph node metastasis, ETE, and advanced N stage (*p* < 0.001) (**Table 1**). Crucially, validation in our independent center cohort (n=155) reinforced this primary association, with Chi-square tests confirming a significant correlation between high MGST1 expression and lymph node metastasis (*p* < 0.001) (**Table 2**). Most importantly, multivariate logistic regression analysis confirmed MGST1 expression as an independent risk factor for LNM in both the TCGA cohort (OR = 1.707, 95% CI: 1.036–2.813, *p* = 0.036) (**Table 3**) and our center’s cohort (OR = 2.168, 95% CI: 1.011–4.649, *p* = 0.047) (**Table 4**), even after adjusting for confounding factors. At the molecular level, high MGST1 expression was associated with a significantly lower Thyroid Differentiation Score (TDS) (**Figure 3M**) and reduced expression of the differentiation marker SLC5A5 (NIS) (**Figure 3N**). Furthermore, directly echoing the immunosuppressive landscape observed in the “Mito-high” subtype, ssGSEA immune profiling demonstrated that MGST1 expression levels strongly dictated the tumor immune microenvironment. Tumors with low MGST1 expression maintained an immune-active state characterized by a significant enrichment of anti-tumor CD8+ T cells, whereas the high-MGST1 group exhibited a marked depletion of these effectors alongside a concurrent elevation in immunosuppressive regulatory T cells (Tregs) (**Figure 3O**).These findings indicate that MGST1 upregulation not only tracks tumor dedifferentiation but also actively orchestrates an immunosuppressive niche, collectively facilitating the aggressive progression observed in LNM-positive patients.

**Table 1:** Relationship between MGST1 expression and clinical features in 499 PTC patients from TCGA database.

	High expression	Low expression	*P*
Total	250	249	
Age			
≥55 years, n (%)	83 (16.7)	82 (16.5)	0.924
<55 years, n (%)	166 (33.3)	167 (33.5)	
Sex			0.544
Male, n (%)	64 (12.9)	70 (14.1)	
Female, n (%)	185 (37.1)	179 (35.9)	
ETE			
Yes, n (%)	96 (20.0)	56 (11.7)	<0.001
No, n (%)	147 (30.6)	181 (37.7)	
LNM			
Yes, n (%)	139 (31.0)	83 (18.5)	<0.001
No, n (%)	89 (19.9)	137 (30.6)	
Histological type			<0.001
Classical/usual, n (%)	188 (38.4)	166 (33.9)	
Follicular, n (%)	31 (6.3)	68 (13.9)	
Tall Cell, n (%)	26 (5.3)	10 (2.0)	
Histological risk			0.004
High, n (%)	30 (6.0)	12 (2.4)	
Low, n (%)	219 (44.0)	237 (47.6)	
N Stage			<0.001
N0, n (%)	89 (22.9)	137 (35.2)	
N1a, n (%)	44 (11.3)	45 (11.6)	
N1b, n (%)	54 (13.9)	20 (5.1)	
T Stage			0.008
T1-T2, n (%)	139 (28.1)	167 (33.7)	
T3-T4, n (%)	109 (22.0)	80 (16.2)	
Focus			0.238
Multifocal, n (%)	119 (24.4)	106 (21.7)	
Unifocal, n (%)	125 (25.6)	138 (28.3)	
BRAF V600E Mutation			0.007
Yes, n (%)	134 (27.3)	103 (21.0)	
No, n (%)	112 (22.9)	141 (28.8)	
Differentiation risk			<0.001
Yes, n (%)	118 (30.4)	74 (19.1)	
No, n (%)	71 (18.3)	125 (32.2)	
TDS, Median (IQR)	-0.547(-1.069, 0.317)	0.357(-0.546, 1.387)	<0.001

**Table 2:** Relationship between MGST1 expression and clinical features in 155 PTC patients from our center.

Clinical features	High expression (n=78)	Low expression (n=77)	P-value
Age (years)			0.349
≥55 years, n (%)	20 (12.9)	25 (16.1)	
<55 years, n (%)	58 (37.4)	52 (33.5)	
Sex			0.220
Male, n (%)	24 (15.5)	17 (11.0)	
Female, n (%)	54 (34.8)	60 (38.7)	
Hashimoto’s Thyroiditis			0.054
Yes, n (%)	4 (2.6)	11 (7.1)	
No, n (%)	74 (47.7)	66 (42.6)	
LNM			0.005
Yes, n (%)	46 (29.7)	28 (18.1)	
No, n (%)	32 (20.6)	49 (31.6)	
BRAF V600E Mutation			0.327
Yes, n (%)	55 (47.0)	51 (43.6)	
No, n (%)	4 (3.4)	7 (6.0)	

**Table 3:** Multivariate logistic regression analysis of risk factors for LNM in the TCGA cohort.

Characteristics	OR	95% CI	*P*
Age (<55 vs. ≥55)	0.474	0.276–0.813	0.007
Sex (Male vs. Female)	1.931	1.108–3.364	0.020
ETE (Yes vs. No)	2.149	0.905–5.106	0.083
MGST1 (High vs. Low)	1.707	1.036–2.813	0.036
T stage (T3–4 vs. T1-2)	1.276	0.562–2.902	0.560
BRAF V600E Mutation (Mut vs. WT)	0.826	0.437–1.562	0.826
Histological type (Tall Cell vs. others)	1.055	0.424–2.626	0.909
TDS	0.604	0.448–0.813	0.001

**Table 4:** Multivariate logistic regression analysis of risk factors for LNM in our center cohort.

Characteristics	OR	95% CI	*P*
Age (<55 vs. ≥55)	0.658	0.283–1.530	0.331
Sex (Male vs. Female)	1.152	0.491–2.703	0.746
Hashimoto’s Thyroiditis (Yes vs. No)	0.620	0.171–2.253	0.468
MGST1 (High vs. Low)	2.168	1.011–4.649	0.047
BRAF V600E Mutation (Mut vs. WT)	1.365	0.366–5.093	0.643

### 3.4 Single-cell trajectory tracing defines MGST1 as a metabolically hyperactive, dedifferentiated metastatic subpopulation at the terminal phase of tumor evolution

**Figure 4:**
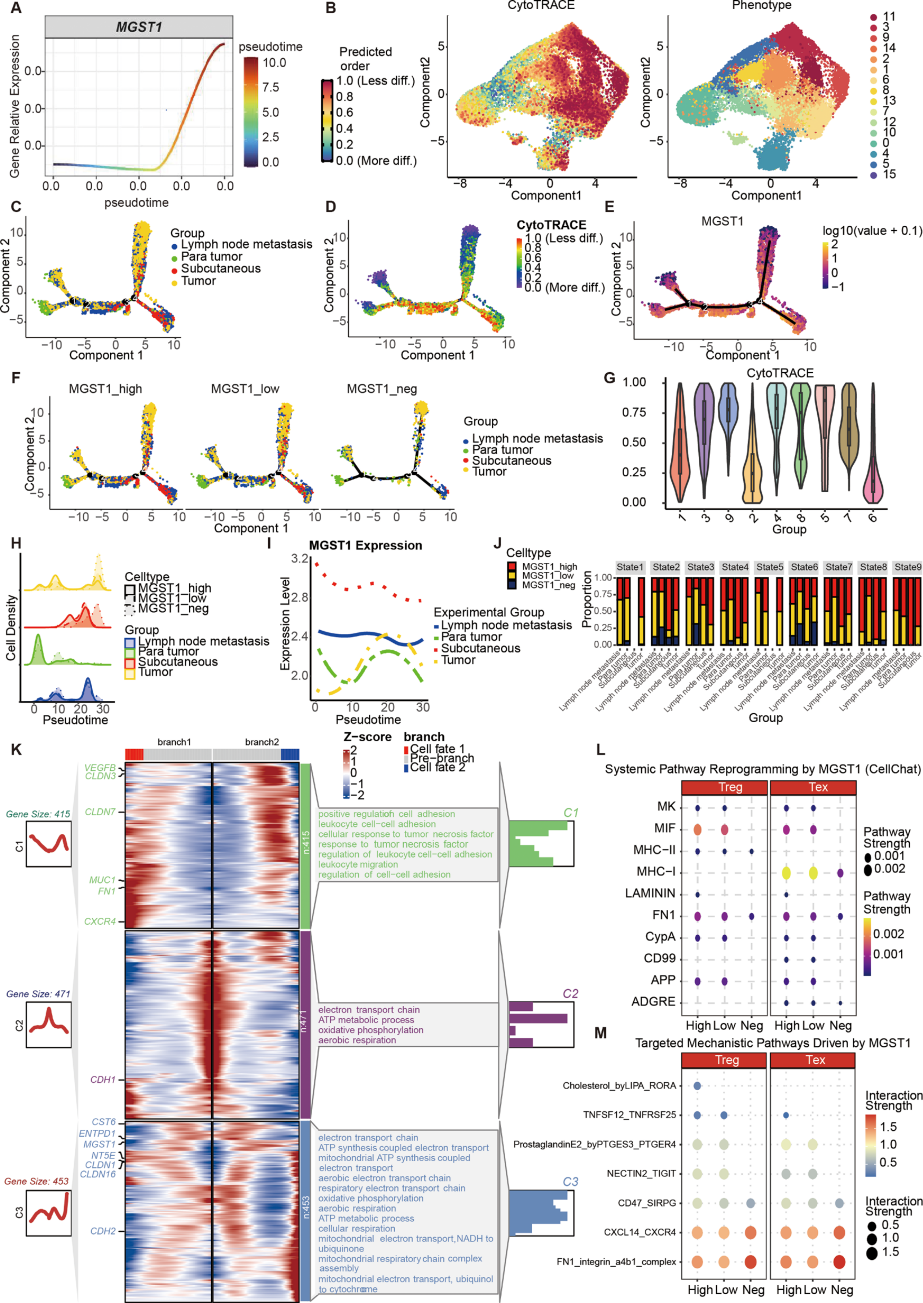
Single-cell trajectory tracing defines MGST1 as a metabolically hyperactive, dedifferentiated metastatic subpopulation at the terminal phase of tumor evolution. **(A)** Pseudotime kinetics plot showing the exponential surge in MGST1 expression specifically at the terminal phase of tumor evolution. **(B)** UMAP embeddings colored by CytoTRACE score (left) and phenotypic clusters (right), visualizing the global differentiation landscape. **(C–E)** Single-cell trajectory trees colored by sample group **(C)**, CytoTRACE score **(D)**, and MGST1 expression **(E)**. Note the precise spatial overlap between the high CytoTRACE score (dedifferentiated status) and MGST1 expression at the trajectory terminus. **(F)** Split-trajectory plots confirm that MGST1-high cells specifically occupy the late-stage evolutionary branch. **(G)** Violin plots of CytoTRACE scores, validating the dedifferentiated, stem-like phenotype of MGST1-high cells. **(H)** Cell density distribution along pseudotime, showing the specific accumulation of MGST1-high cells in the late phase. **(I)** Comparative pseudotime kinetics of MGST1 expression across sample groups, highlighting sustained high expression in metastasis-associated groups (Subcutaneous and LNM) compared to primary tumors. **(J)** Stacked bar plot showing the compositional enrichment of MGST1-high cells within metastasis-associated states. **(K)** BEAM heatmap comparing transcriptional programs between branches. The MGST1-high branch (Branch 2) is characterized by the upregulation of mesenchymal markers (e.g., CDH2) and hyperactivation of mitochondrial bioenergetic pathways (e.g., oxidative physics, electron transport chain), in contrast to the cell adhesion-enriched Branch 1. **(L)** Dot plot of CellChat analysis showing the specific enrichment of immunosuppressive signaling pathways (e.g., MIF, APP, and CypA) from MGST1-expressing cells to Tregs and Tex. **(M)** Dot plot of CellPhoneDB analysis detailing the receptor-ligand interactions, including the CD47-SIRPG axis and metabolic-immune checkpoints (PGE2 and NECTIN2-TIGIT), between MGST1-expressing cells and immune subsets.

Leveraging single-cell RNA sequencing (scRNA-seq) to dissect cellular heterogeneity, we reconstructed the developmental trajectory of tumor cells. Pseudotime analysis revealed a striking temporal lag: MGST1 expression, which was quiescent during early developmental phases, exhibited a sharp, exponential surge at the terminal end of the trajectory (**Figure 4A**). This pseudotime-dependent pattern was robustly corroborated by bulk RNA-seq trajectory analysis (**Supplementary Figure 3**).

Cellular annotation localized this signal predominantly to the Thyrocyte lineage (**Supplementary Figures 4**–**S5**). To dissect the differentiation status of these cells, we first characterized the global differentiation landscape. Mapping of CytoTRACE scores onto the UMAP embedding (**Figure 4B**) visualized a continuous gradient from differentiated to dedifferentiated states, while phenotypic clustering (**Figure 4C**) further delineated cellular heterogeneity. To verify the spatiotemporal relationship between MGST1 and dedifferentiation, we projected these features onto the pseudotime trajectory tree. The mapping of CytoTRACE scores on the trajectory (**Figure 4D**) clearly defined the differentiation gradient, identifying the trajectory terminus as the state with the highest differentiation potential. Strikingly, the MGST1 feature plot (**Figure 4E**) revealed a precise spatial overlap with **Figure 4D**, showing that MGST1 expression was strictly confined to this dedifferentiated terminus. Stratification by MGST1 status confirmed that MGST1-high cells occupied the late-stage evolutionary branch (**Figure 4F**). Quantitative analysis further showed that these cells had significantly higher CytoTRACE scores, indicative of greater stemness and dedifferentiation, than cells in other groups (**Figure 4G**). Spatiotemporal analysis revealed that the accumulation of MGST1-high cells occurred exclusively in late evolutionary phases (**Figure 4H*****)***. Notably, comparative trajectory analysis demonstrated that metastasis-associated groups (Subcutaneous and LNM) consistently maintained significantly elevated MGST1 expression levels throughout the trajectory compared to primary tumors (**Figure 4I**), linking MGST1 upregulation to metastatic potential. Compositional analysis confirmed the disproportionate enrichment of this subpopulation in metastasis-associated states (**Figure 4J**), substantiating their role as the seed population for colonization.

Mechanistically, BEAM deciphered the molecular machinery empowering this subpopulation (**Figure 4K**). In sharp contrast to the early branch (Branch 1), which retained cell adhesion signatures (e.g., CLDN7, MUC1), the MGST1-high branch (Branch 2) underwent fundamental transcriptional reprogramming, driving the epithelial-to-mesenchymal transition (EMT). This state was characterized not only by the loss of the epithelial marker CDH1 and gain of the mesenchymal marker CDH2, but also by the specific hyperactivation of mitochondrial bioenergetic pathways, including oxidative phosphorylation and the electron transport chain.

Concurrently, intercellular communication analyses revealed that this subpopulation actively orchestrates an immunosuppressive microenvironment. CellChat analysis showed that the MIF, APP, and CypA signaling pathways, which target regulatory T cells (Tregs) and exhausted T cells (Tex), were highly active in MGST1-expressing cells but absent in MGST1-negative cells (**Figure 4L**). At the receptor-ligand level, CellPhoneDB confirmed that MGST1-positive cells specifically established the anti-phagocytic CD47-SIRPG axis and triggered critical checkpoints, including Prostaglandin E2 (PGE2) and NECTIN2-TIGIT (**Figure 4M**). These findings mirror the metabolic and immune architecture of the “Mito-high” subtype at single-cell resolution, confirming that MGST1 marks an aggressive subpopulation that leverages mitochondrial plasticity and immune evasion to drive dedifferentiation and metastasis.

### 3.5 Pharmacological targeting of MGST1 via toxoflavin abrogates malignant phenotypes and demonstrates strict target dependency

**Figure 5:**
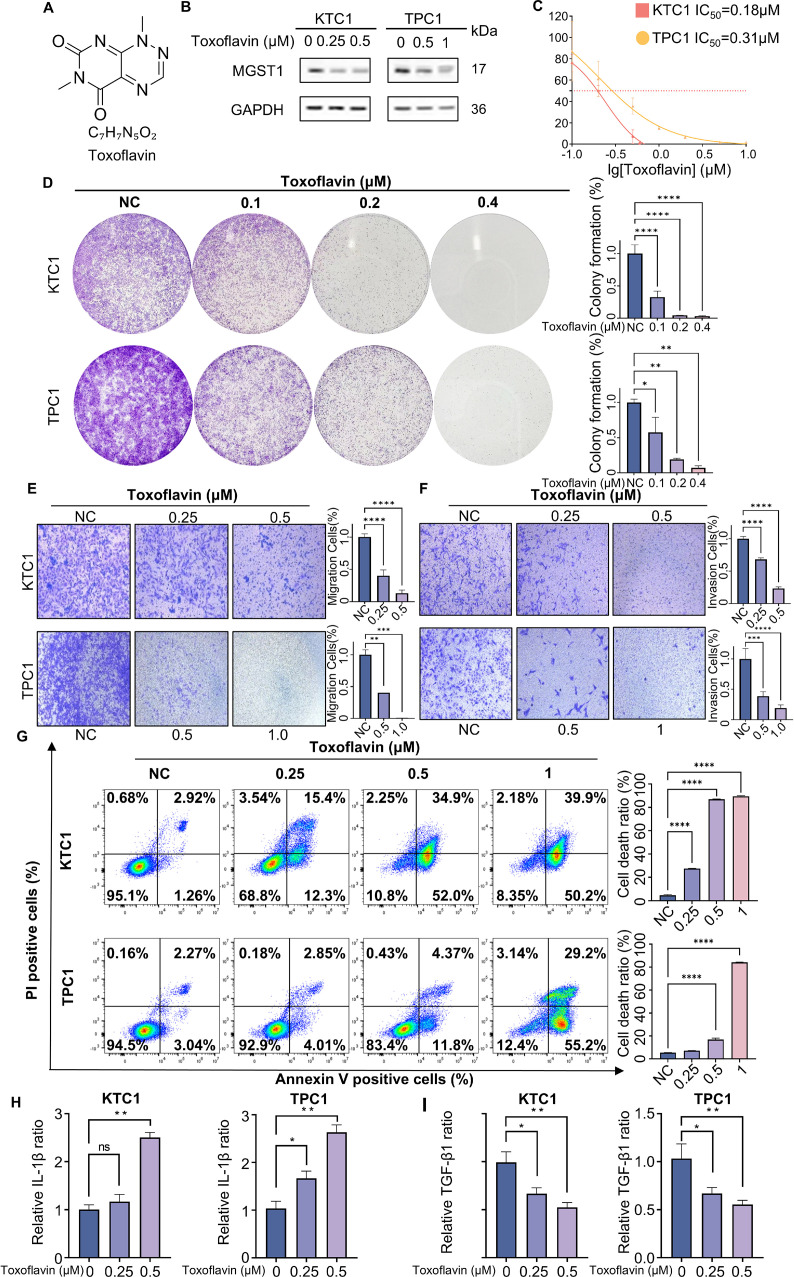
Pharmacological targeting of MGST1 via toxoflavin abrogates malignant phenotypes. **(A)** Chemical structure of the small-molecule inhibitor Toxoflavin. **(B)** Western blot analysis demonstrates a dose-dependent reduction in MGST1 protein levels in KTC1 and TPC1 cells upon Toxoflavin treatment. **(C)** Drug sensitivity curves showing potent cytotoxicity with calculated IC50 values. **(D)** Colony formation assays revealed that Toxoflavin treatment nearly abrogates the clonogenic survival of PTC cells. **(E, F)** Representative images (left) and quantification (right) of Transwell assays showing dose-dependent inhibition of cell migration **(E)** and invasion **(F)**. **(G)** Flow cytometry analysis quantifying the induction of apoptosis (Annexin V^+^ cells) following Toxoflavin treatment, confirming the mechanism of growth suppression. **(H)** Levels of IL-1β in cell culture supernatants detected by ELISA. **(I)** Levels of TGF-β1 in cell culture supernatants detected by ELISA. ^*^P < 0.05, ^**^P < 0.01, ^***^P < 0.001, ^****^P < 0.0001.

Given the established role of MGST1 as a key driver of LNM and dedifferentiation, we evaluated the therapeutic efficacy of Toxoflavin, a small-molecule inhibitor targeting this protein. Toxoflavin treatment elicited a potent, dose-dependent ablation of MGST1 protein levels in both KTC1 and TPC1 cells (**Figures 5A, B**). This pharmacological blockade translated into profound cytotoxicity (IC_50_: 0.18 µM for KTC1; 0.31 µM for TPC1) (**Figure 5C**) and nearly abrogated clonogenic survival (**Figure 5D**). Functionally, Toxoflavin treatment dismantled the invasive and migratory potential of tumor cells in a dose-dependent manner (**Figures 5E, F**), a suppression mechanistically driven by the robust induction of apoptosis (**Figure 5G**). Moreover, Pharmacological inhibition of MGST1 promotes the release of inflammatory cytokine IL-1β (**Figure 5H**) and inhibits the release of immunosuppressive cytokine TGF-β1(**Figure 5I**).

**Figure 6:**
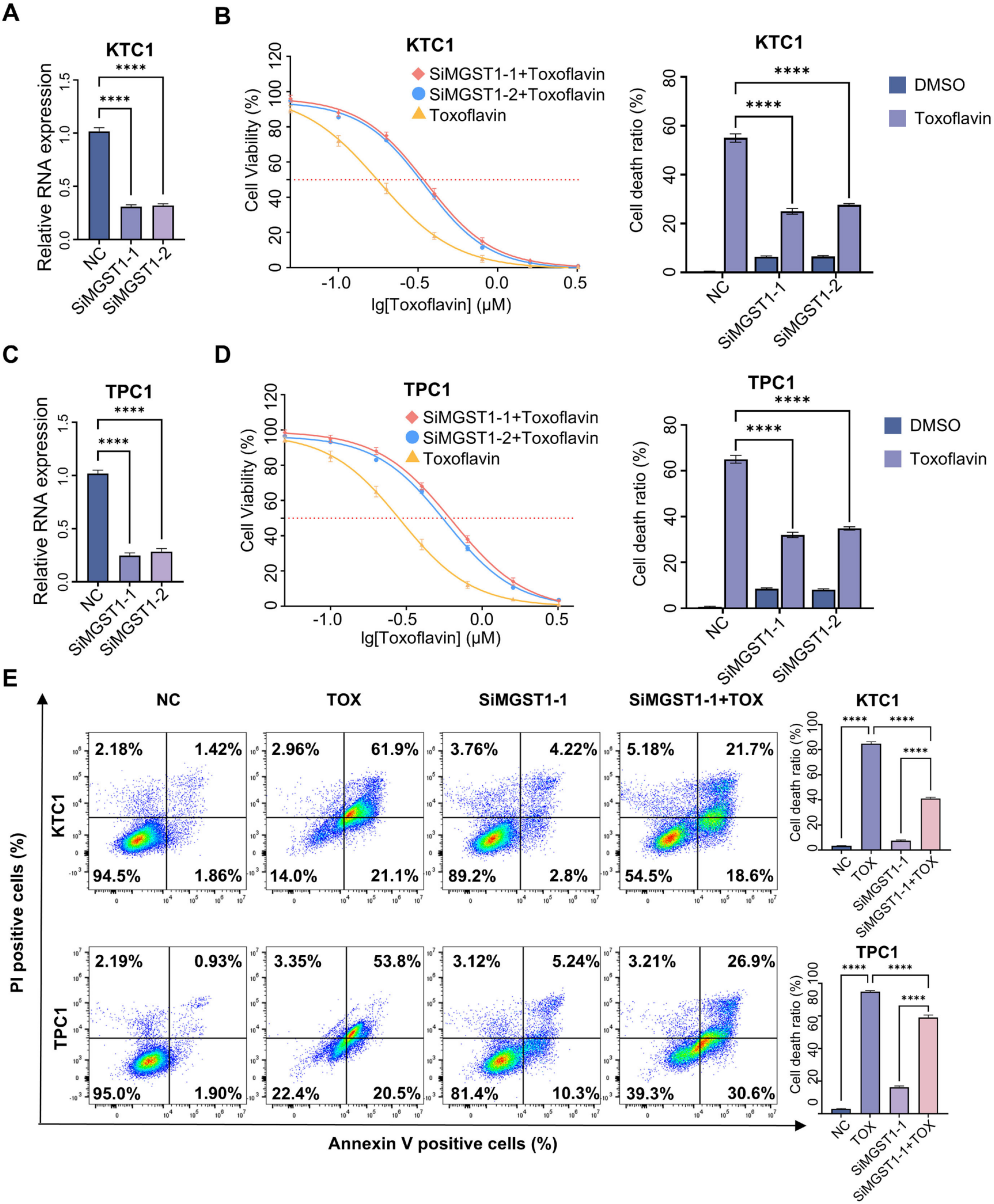
Target specificity validation demonstrates that MGST1 depletion confers resistance to toxoflavin. **(A, C)** Quantitative real-time PCR (qPCR) validating the significant knockdown efficiency of MGST1-specific siRNAs (SiMGST1–1 and SiMGST1-2) in KTC1 **(A)** and TPC1 **(C)** cells. **(B, D)** Dose-response survival curves (left) and cell death quantification (right) for KTC1 **(B)** and TPC1 **(D)** cells. Note the distinct “phenotypic rescue” effect, where MGST1-silenced cells exhibit a parallel rightward shift in survival curves and significantly reduced cell death rates compared to the negative control (NC) group upon Toxoflavin exposure. **(E)** Representative flow cytometry plots (left) and quantification (right) of apoptosis (Annexin V/PI staining). MGST1 depletion significantly attenuates Toxoflavin (1 μM)-induced apoptosis in both KTC1 and TPC1 cells, confirming that the drug’s cytotoxicity depends on MGST1 expression. ^****^P < 0.0001.

To rigorously validate target specificity, we performed loss-of-function assays using siRNA-mediated depletion (**Figures 6A, C**). Drug sensitivity analysis revealed a distinct “phenotypic rescue” effect: in sharp contrast to the highly sensitive control cells, MGST1-silenced cells exhibited significant resistance to Toxoflavin-induced cytotoxicity. This target dependency was evidenced by a parallel rightward shift in survival curves (**Figures 6B, D**, Left panels) and a remarkable attenuation of cell death rates at identical drug concentrations (0.2 µM for KTC1; 0.4 µM for TPC1) (**Figures 6B, D**, Right panels). Flow cytometry further corroborated these findings, showing that the apoptotic fraction was significantly lower in the si-MGST1 group compared to the control group upon Toxoflavin exposure (**Figure 6E**). Collectively, these results provide compelling evidence that MGST1 serves as the critical molecular target for Toxoflavin, and its depletion confers resistance to drug-induced oxidative death, confirming the high specificity of this pharmacological intervention.

## 4 Discussion

LNM is a common occurrence in the progression of PTC and is strongly associated with locoregional recurrence and poor prognosis (4–6). Approximately 40%–60% of patients with PTC present with LNM at diagnosis, underscoring the importance of elucidating the molecular determinants of LNM to improve clinical outcomes (7–9). In this study, by integrating multi-omics data, network analysis, pseudotime trajectory modeling, and machine learning, we identified a subset of mitochondria-related genes significantly correlated with LNM. Among them, MGST1 emerged as a hub gene consistently overexpressed in metastatic tissues and strongly associated with advanced TNM stage and poor survival, suggesting its central role in promoting lymphatic dissemination in PTC. In addition to metabolic function, the identification of MGST1 as a key driving factor emphasizes that mitochondrial metabolic reprogramming and tumor immune microenvironment (TIME) remodeling are hallmarks that jointly promote metastasis. In the context of PTC, the transition to a metastatic phenotype likely requires both metabolic adaptation to the lymphatic environment and the active suppression of local immune surveillance.

MGST1 encodes microsomal glutathione S-transferase 1, a key enzyme that regulates glutathione metabolism and redox homeostasis (22–24). Elevated MGST1 expression has been linked to enhanced proliferation, invasion, and poor prognosis in multiple cancers, including gastric cancer and melanoma (25–28). Mechanistically, MGST1 modulates PI3K/AKT/mTOR signaling and has been shown to inhibit ferroptosis, a regulated cell death pathway increasingly recognized as a driver of tumor progression and therapeutic resistance (29, 30). Crucially, ferroptosis transcends being a simple cell-autonomous event; it serves as a potent trigger for immune responses (31, 32). The inhibition of ferroptosis by MGST1 potentially thwarts the liberation of damage-associated molecular patterns (DAMPs) and lipid peroxides, thereby impeding the subsequent activation of dendritic cells and CD8+ T cells. Consequently, high MGST1 expression might facilitate an “immune-cold” phenotype in PTC, allowing metastatic cells to evade immune detection during their transit through the lymphatics. Our findings indicated that MGST1 may contribute not only to PTC metastasis but also to therapy resistance, particularly in iodine-refractory PTC, where treatment options are limited. Notably, sorafenib, one of the main systemic therapies for refractory thyroid cancer, is a ferroptosis inducer (33, 34). Resistance to sorafenib remains a major clinical challenge. The roles of mitochondrial metabolic defense and immune evasion suggest that MGST1-mediated resistance is dual-faceted: it protects the cell from drug-induced ferroptosis while simultaneously maintaining a suppressive immune barrier. Based on our data, we propose that pharmacological inhibition of MGST1 could restore ferroptosis sensitivity and resensitize iodine-refractory PTC to sorafenib, providing a rational combinatorial therapeutic strategy for future translational studies.

Functionally, our *in vitro* experiments confirmed the oncogenic role of MGST1 in PTC progression. Pharmacological inhibition of MGST1 with toxoflavin markedly reduced colony formation, migration, and invasion of KTC1 and TPC1 cells and significantly promoted apoptosis. Moreover, Pharmacological inhibition of MGST1 promotes the release of inflammatory cytokine IL-1β and inhibits the release of immunosuppressive cytokine TGF-β1. Both IL-1β and TGF-β1 are well-established immune-related modulators that play pivotal roles in shaping the tumor microenvironment (35, 36). MGST1-silenced cells exhibited significant resistance to Toxoflavin-induced cytotoxicity. All these results indicate that MGST1 has the potential to suppress immunity, promote tumor growth, and metastasis. Our multi-omics and machine learning findings provide convergent evidence that MGST1 is not only a late-activated mitochondrial driver but also a promising biomarker for LNM risk stratification and a viable therapeutic target for PTC.

Despite these insights, limitations exist. First and foremost, our study is primarily a clinical exploration aimed at finding a novel prognostic biomarker, rather than a mechanistic investigation designed to solve a specific biological problem or test a defined causal model. Consequently, our multi-omics pipeline serves primarily as a hypothesis-generating framework. The observations from our bioinformatic and single-cell trajectory analyses—such as the positioning of MGST1 and its proposed links to EMT, mitochondrial metabolic reprogramming, and the “immune-cold” phenotype—are essentially coincidental discoveries encountered during the biomarker screening process. We merely report these phenomena as correlative associations without establishing definitive lineage direction or functional causality. Specifically, our immunological conclusions rely predominantly on computational estimations. Due to current constraints in research scope and funding, we did not perform *in vivo* lineage tracing, tumor-immune cell co-culture assays, or immune perturbation studies. Therefore, the precise molecular mechanisms by which MGST1 promotes LNM remain to be elucidated. Furthermore, the dynamic interactions between MGST1-expressing tumor cells and specific immune subsets, such as myeloid-derived suppressor cells (MDSCs) or Tregs, remain to be fully mapped. The potential of MGST1 inhibition to overcome sorafenib resistance warrants further validation in preclinical models that account for a functional immune system. Future studies utilizing robust *in vivo* models and strict causal experimental designs are needed to transition these coincidental findings from correlative observations to causal demonstrations.

## 5 Conclusions

In summary, our study provides evidence that MGST1, as a mitochondrial driver, promotes immune evasion and metabolic reprogramming in PTC, thereby facilitating LNM. Beyond its prognostic value, MGST1 represents a viable pharmacological target whose inhibition may suppress metastasis and address therapeutic resistance. Future preclinical and clinical studies should evaluate MGST1 inhibitors, alone or in combination with sorafenib, as a novel strategy to improve outcomes for patients with iodine-refractory PTC by simultaneously targeting metabolic vulnerabilities and restoring the tumor’s immunogenicity.

## Data Availability

The original contributions presented in the study are included in the article/**Supplementary Material**. Further inquiries can be directed to the corresponding author/s.

## Supplementary material

Supplementary Figure 1 Data preprocessing and determination of robust mitochondrial molecular subtypes. **(A, B)** Principal Component Analysis (PCA) plots visualizing the data structure before and after processing. **(A)** Distribution of raw samples from GTEx and TCGA databases. **(B)** Clear separation between tumor and normal samples following data integration and batch correction. **(C)** t-SNE plot illustrating the distribution of samples based on Lymph Node Metastasis (LNM) status. **(D, E)** Identification of the optimal cluster number for Non-negative Matrix Factorization (NMF). **(D)** The consensus clustering matrix for k=2 displays distinct, robust block structures. **(E)** Comprehensive evaluation of factorization ranks (k=2 to 7) using multiple metrics (cophenetic correlation, dispersion, silhouette scores, etc.), confirming k=2 as the optimal number of clusters. **(F)** A Venn diagram illustrates the multi-step intersection strategy (including tumor-specific DEGs, LNM-associated DEGs, and mitochondrial genes) to screen for candidate driver genes. **(G)** t-SNE plot validating the distinct spatial separation of the identified Class 1 and Class 2 subtypes based on the final gene signature.

Supplementary Figure 2 Weighted gene co-expression network analysis (WGCNA) independently prioritizes the MGST1-containing module as a key driver of lymph node metastasis. **(A)** A sample clustering dendrogram and trait heatmap were constructed to detect outliers and visualize the distribution of clinical traits. **(B, C)** Analysis of network topology for soft-thresholding power (β) selection, illustrating the mean connectivity **(B)** and the scale-free topology fit index **(C)** across different power values. **(D)** Hierarchical clustering dendrogram of differentially expressed genes based on the topological overlap measure (TOM) dissimilarity, with assigned module colors indicated below the tree. **(E)** Heatmap quantifying the correlation between module eigengenes and clinical traits. The ME3 module exhibits the strongest positive correlation with lymph node metastasis (R = 0.35, *P* < 9×10^-15^) and serves as the host module for MGST1.

Supplementary Figure 3 Bulk RNA-seq trajectory analysis corroborates the late-stage activation of MGST1 and the evolutionary continuum of mitochondrial subtypes. **(A)** Sample distance matrix visualizing the global transcriptomic dissimilarity between Normal, Mito-low, and Mito-high groups. **(B)** Pseudotime trajectory inferred from bulk RNA-seq data, delineating a continuous evolutionary path from Normal tissues through the Mito-low subtype to the terminal Mito-high subtype. **(C–J)** Heatmaps characterizing gene expression kinetics along the inferred pseudotime timeline across different molecular orders (Order 1–4). Notably, consistent with single-cell findings, MGST1 is identified within the late-response gene clusters **(E, I, J)**, exhibiting a sharp expression surge specifically in the “Mito-high” phase (timeline terminus), thereby validating its role as a marker of advanced tumor progression.

Supplementary Figure 4 Comprehensive annotation and functional characterization of the single-cell transcriptomic landscape. **(A)** t-SNE embedding visualizing the eight major cell lineages identified in the scRNA-seq dataset, accompanied by a summary table detailing canonical marker genes and cell composition across different groups. **(B)** Violin plots validate the expression specificity of the canonical markers used for cell-type identification. **(C)** Functional enrichment analysis summarizing the distinct biological roles associated with each identified cell cluster. **(D)** Feature plots illustrating the spatial distribution and expression intensity of key lineage markers on the t-SNE map.

Supplementary Figure 5 Single-cell resolution identifies MGST1 as a thyrocyte-specific driver gene significantly upregulated in lymph node metastasis. **(A)** Jitter plot displaying the top differentially expressed genes (DEGs) across eight identified cell clusters. MGST1 is identified as a top marker gene specifically enriched in the Thyrocyte lineage. **(B)** Dot plot validating the expression specificity of target genes (PMAIP1, SFXN3, and MGST1), confirming that MGST1 is predominantly expressed in Thyrocytes. (C–E) t-SNE feature plots visualizing the spatial distribution and expression intensity of MGST1 **(C)**, PMAIP1 **(D)**, and SFXN3 **(E)**. **(F–H)** Violin plots quantifying the expression levels of these genes across different sample groups (normal, non-LNM, LNM, and others). **(I)** Split t-SNE plots comparing MGST1 expression across sample groups, illustrating its spatial expansion and high expression intensity specifically in LNM and metastatic lesions. **(J)** Pseudotime trajectory tree colored by pseudotime units, visualizing the continuous developmental path of tumor cells used for trajectory analysis. ^****^*P* < 0.0001.

Supplementary Figure 6 Uncropped western blot images. Original, uncropped blots for MGST1 and GAPDH shown in **Figure 5B**.
