# Supplementary figures and images for: MGST1 drives lymph node metastasis in papillary thyroid carcinoma via mitochondrial metabolic reprogramming and immune suppression
Source: Front Immunol. 2026 Jun 4;17:1848083. doi: 10.3389/fimmu.2026.1848083 (PMC13275704; doi:10.3389/fimmu.2026.1848083)

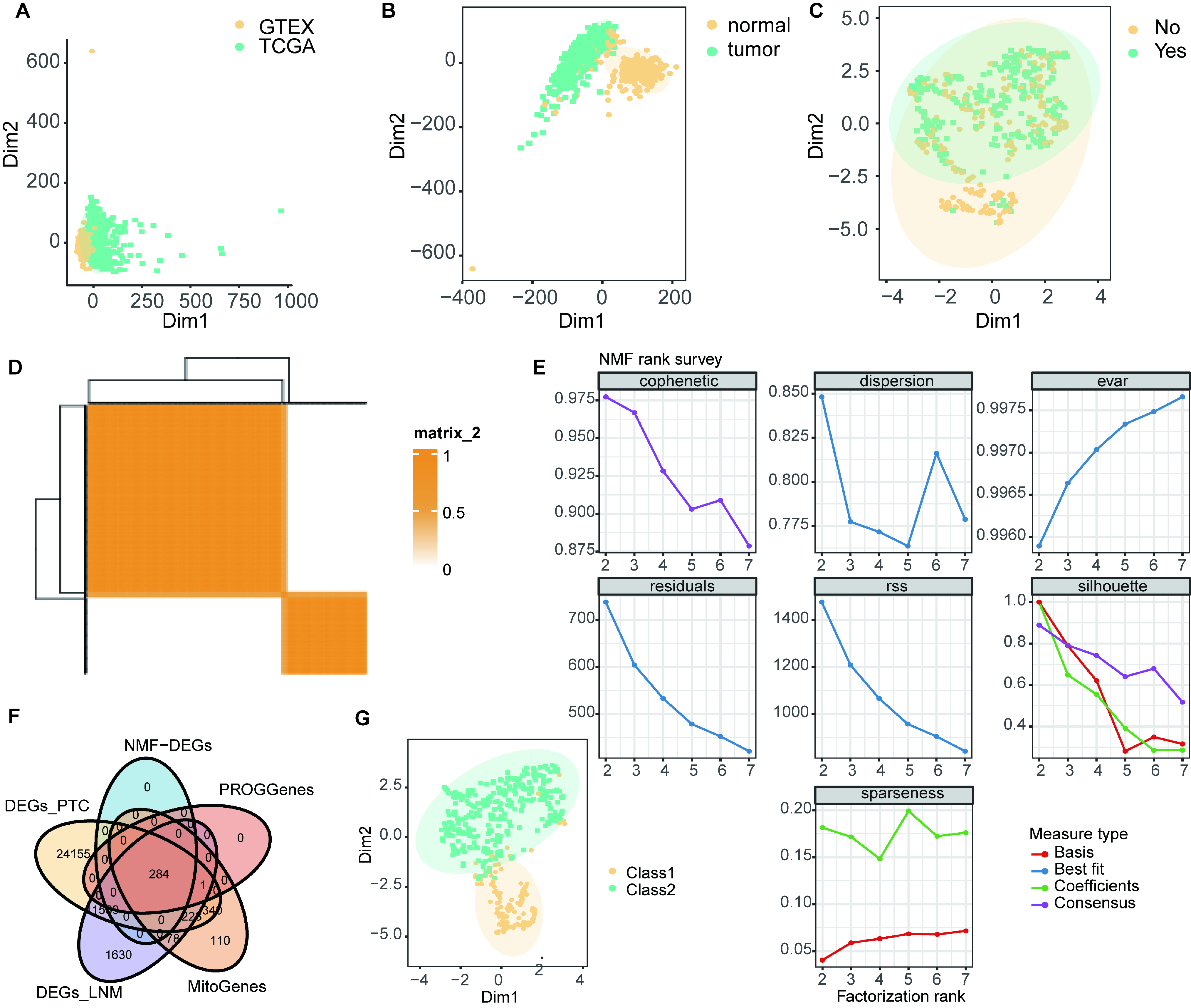

Supplement: Supplementary Figure 1 — Data preprocessing and determination of robust mitochondrial molecular subtypes. (A, B) Principal Component Analysis (PCA) plots visualizing the data structure before and after processing. (A) Distribution of raw samples from GTEx and TCGA databases. (B) Clear separation between tumor and normal samples following data integration and batch correction. (C) t-SNE plot illustrating the distribution of samples based on Lymph Node Metastasis (LNM) status. (D, E) Identification of the optimal cluster number for Non-negative Matrix Factorization (NMF). (D) The consensus clustering matrix for k=2 displays distinct, robust block structures. (E) Comprehensive evaluation of factorization ranks (k=2 to 7) using multiple metrics (cophenetic correlation, dispersion, silhouette scores, etc.), confirming k=2 as the optimal number of clusters. (F) A Venn diagram illustrates the multi-step intersection strategy (including tumor-specific DEGs, LNM-associated DEGs, and mitochondrial genes) to screen for candidate driver genes. (G) t-SNE plot validating the distinct spatial separation of the identified Class 1 and Class 2 subtypes based on the final gene signature. [file Image1.jpeg]

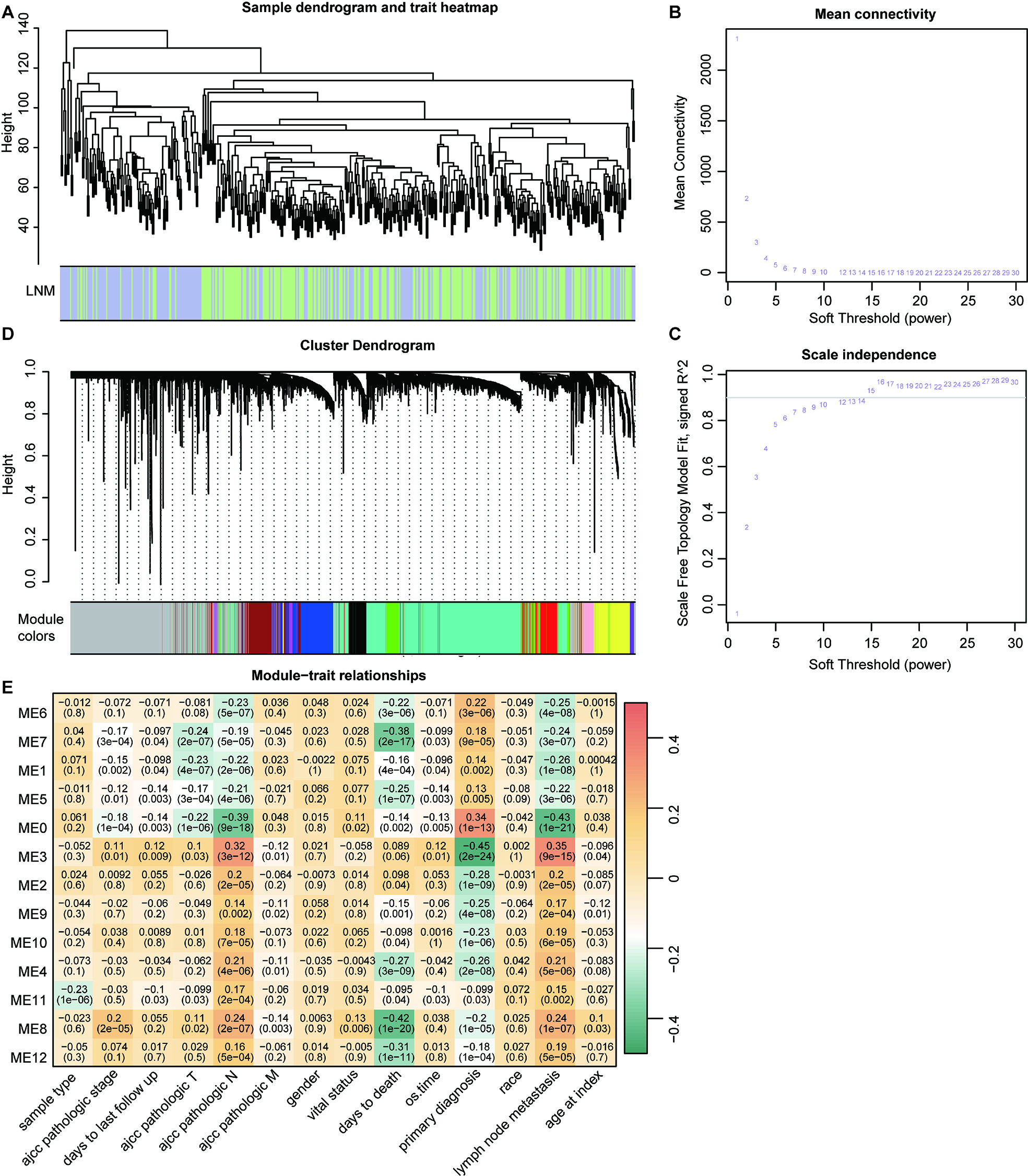

Supplement: Supplementary Figure 2 — Weighted gene co-expression network analysis (WGCNA) independently prioritizes the MGST1-containing module as a key driver of lymph node metastasis. (A) A sample clustering dendrogram and trait heatmap were constructed to detect outliers and visualize the distribution of clinical traits. (B, C) Analysis of network topology for soft-thresholding power (β) selection, illustrating the mean connectivity (B) and the scale-free topology fit index (C) across different power values. (D) Hierarchical clustering dendrogram of differentially expressed genes based on the topological overlap measure (TOM) dissimilarity, with assigned module colors indicated below the tree. (E) Heatmap quantifying the correlation between module eigengenes and clinical traits. The ME3 module exhibits the strongest positive correlation with lymph node metastasis (R = 0.35, P < 9×10^-15^) and serves as the host module for MGST1. [file Image2.jpeg]

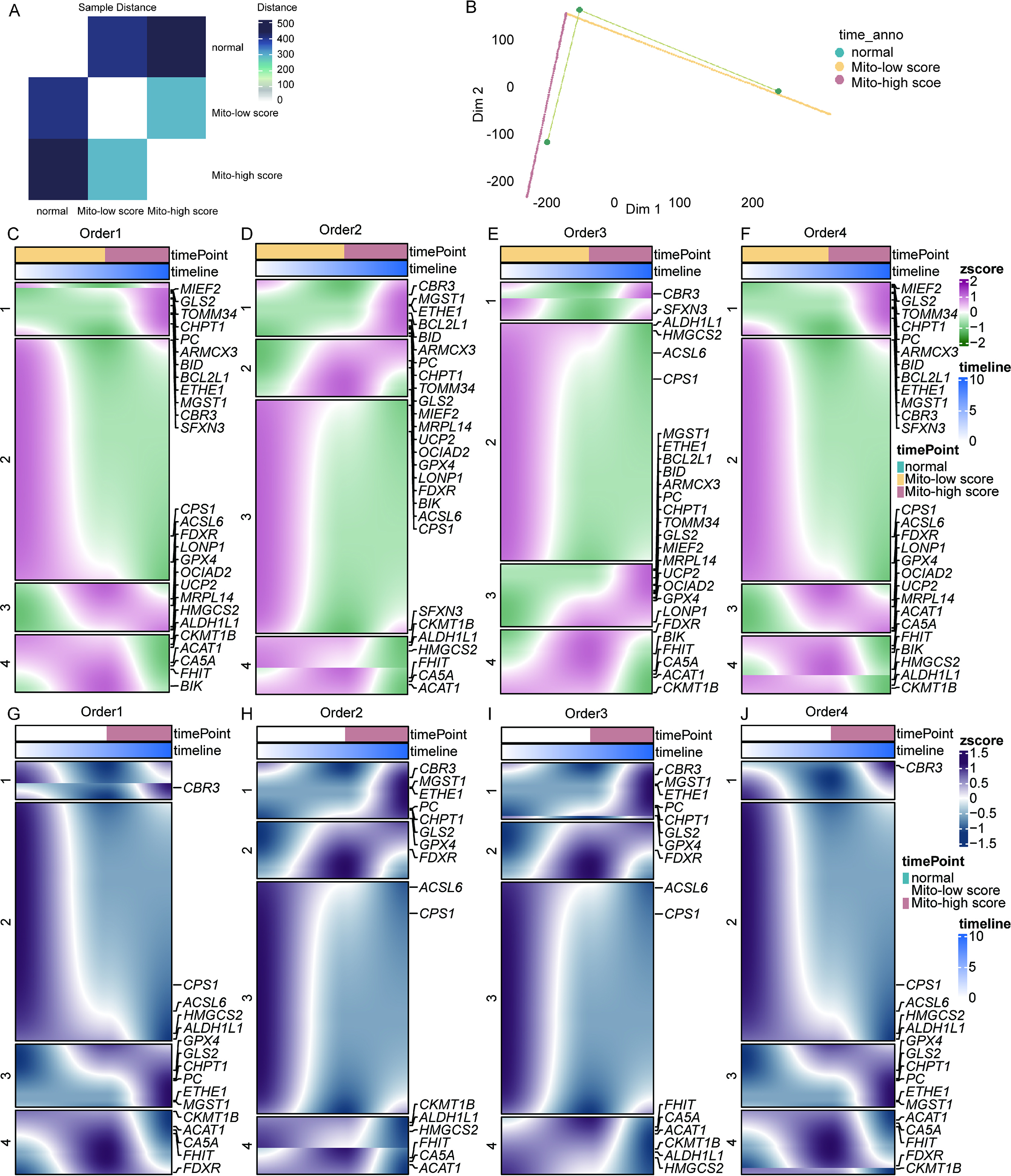

Supplement: Supplementary Figure 3 — Bulk RNA-seq trajectory analysis corroborates the late-stage activation of MGST1 and the evolutionary continuum of mitochondrial subtypes. (A) Sample distance matrix visualizing the global transcriptomic dissimilarity between Normal, Mito-low, and Mito-high groups. (B) Pseudotime trajectory inferred from bulk RNA-seq data, delineating a continuous evolutionary path from Normal tissues through the Mito-low subtype to the terminal Mito-high subtype. (C–J) Heatmaps characterizing gene expression kinetics along the inferred pseudotime timeline across different molecular orders (Order 1–4). Notably, consistent with single-cell findings, MGST1 is identified within the late-response gene clusters (E, I, J), exhibiting a sharp expression surge specifically in the “Mito-high” phase (timeline terminus), thereby validating its role as a marker of advanced tumor progression. [file Image3.jpeg]

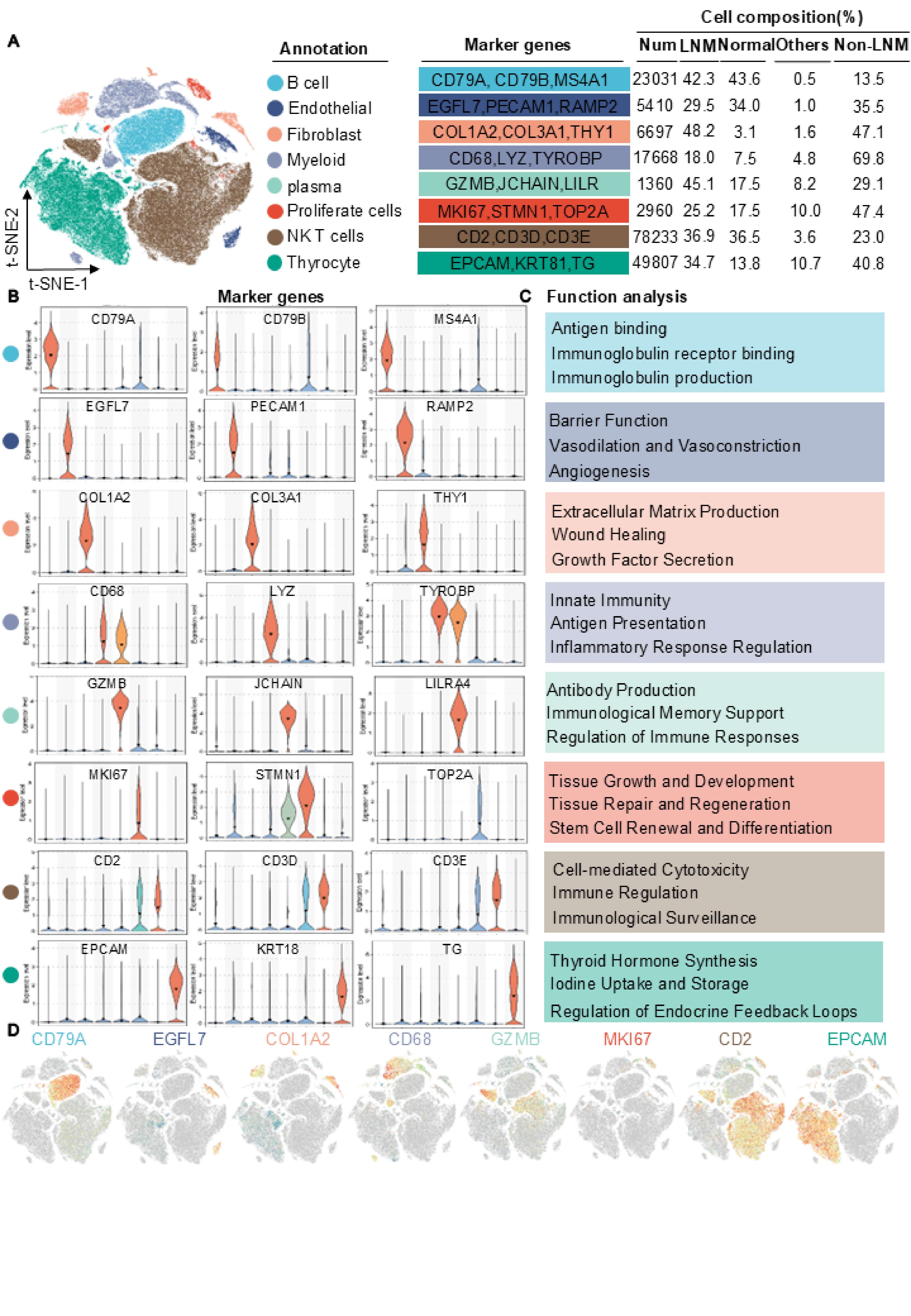

Supplement: Supplementary Figure 4 — Comprehensive annotation and functional characterization of the single-cell transcriptomic landscape. (A) t-SNE embedding visualizing the eight major cell lineages identified in the scRNA-seq dataset, accompanied by a summary table detailing canonical marker genes and cell composition across different groups. (B) Violin plots validate the expression specificity of the canonical markers used for cell-type identification. (C) Functional enrichment analysis summarizing the distinct biological roles associated with each identified cell cluster. (D) Feature plots illustrating the spatial distribution and expression intensity of key lineage markers on the t-SNE map. [file Image4.jpeg]

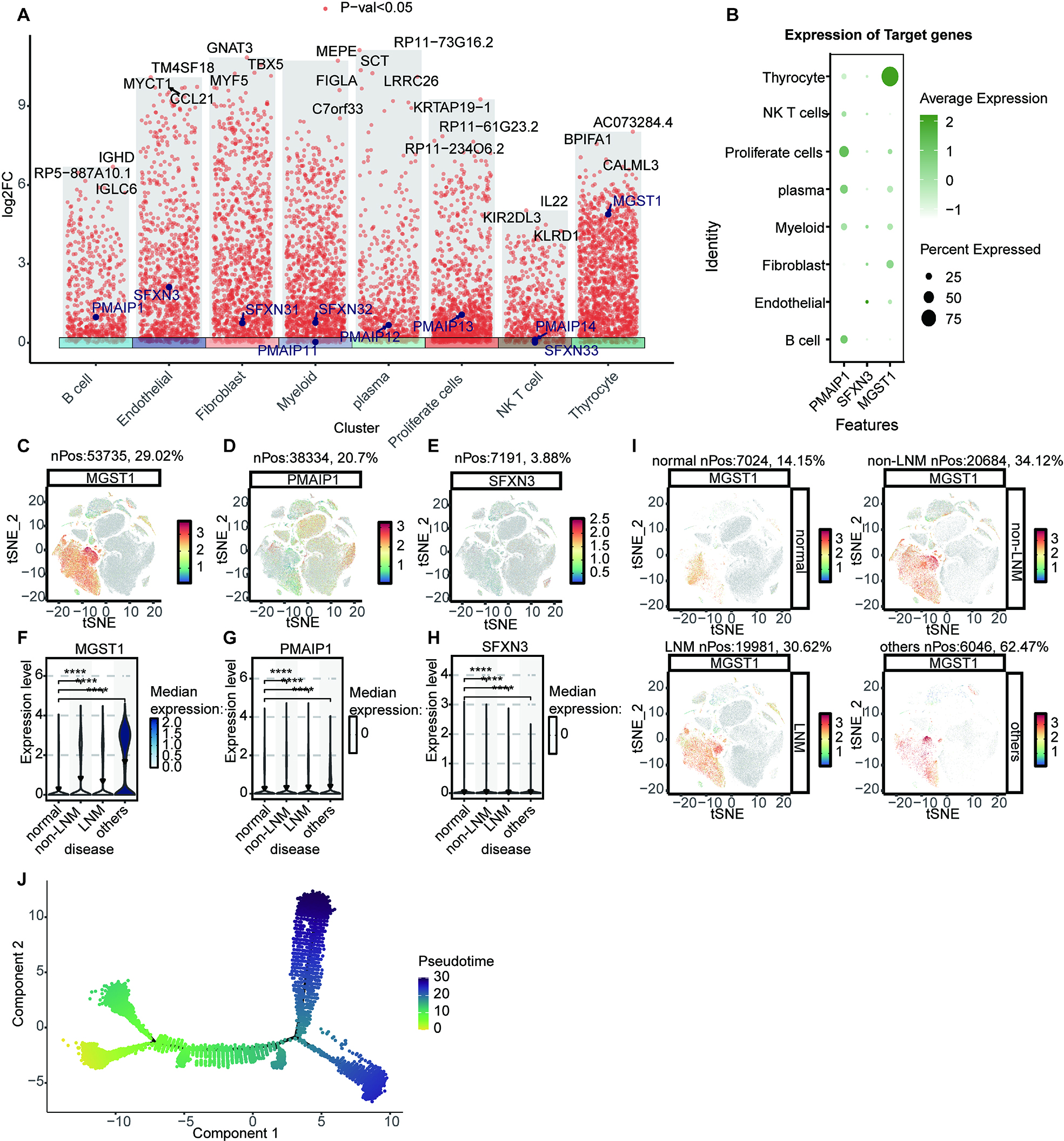

Supplement: Supplementary Figure 5 — Single-cell resolution identifies MGST1 as a thyrocyte-specific driver gene significantly upregulated in lymph node metastasis. (A) Jitter plot displaying the top differentially expressed genes (DEGs) across eight identified cell clusters. MGST1 is identified as a top marker gene specifically enriched in the Thyrocyte lineage. (B) Dot plot validating the expression specificity of target genes (PMAIP1, SFXN3, and MGST1), confirming that MGST1 is predominantly expressed in Thyrocytes. (C–E) t-SNE feature plots visualizing the spatial distribution and expression intensity of MGST1 (C), PMAIP1 (D), and SFXN3 (E). (F–H) Violin plots quantifying the expression levels of these genes across different sample groups (normal, non-LNM, LNM, and others). (I) Split t-SNE plots comparing MGST1 expression across sample groups, illustrating its spatial expansion and high expression intensity specifically in LNM and metastatic lesions. (J) Pseudotime trajectory tree colored by pseudotime units, visualizing the continuous developmental path of tumor cells used for trajectory analysis. ****P < 0.0001. [file Image5.jpeg]

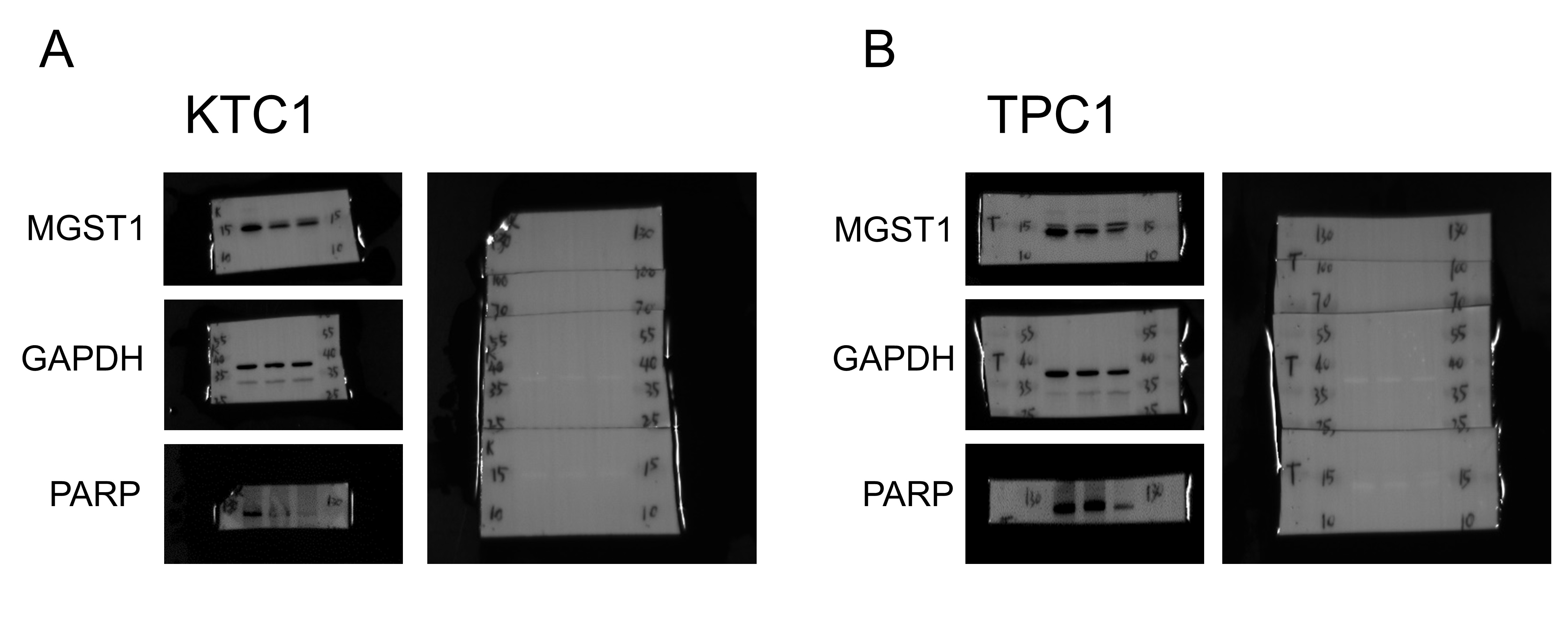

Supplement: Supplementary Figure 6 — Uncropped western blot images. Original, uncropped blots for MGST1 and GAPDH shown in Figure 5B. [file Image6.jpeg]
