# Supplementary material for: MGST1 drives lymph node metastasis in papillary thyroid carcinoma via mitochondrial metabolic reprogramming and immune suppression
Source: Front Immunol. 2026 Jun 4;17:1848083. doi: 10.3389/fimmu.2026.1848083 (PMC13275704; doi:10.3389/fimmu.2026.1848083)
Supplement: Supplementary file 7 [file Table1.docx]

Supplementary Table S2: Association of MGST1 expression with central and lateral compartment lymph node metastasis in the TCGA and in-house cohorts.

|  | High expression | Low expression | *P* |
| --- | --- | --- | --- |
| TCGA |  |  | 0.003 |
| N1a | 44 | 45 |  |
| N1b | 54 | 21 |  |
| Inhouse cohort |  |  | 0.723 |
| N1a | 33 | 19 |  |
| N1b | 13 | 9 |  |
