# Supplementary material for: MGST1 drives lymph node metastasis in papillary thyroid carcinoma via mitochondrial metabolic reprogramming and immune suppression
Source: Front Immunol. 2026 Jun 4;17:1848083. doi: 10.3389/fimmu.2026.1848083 (PMC13275704; doi:10.3389/fimmu.2026.1848083)
Supplement: Supplementary file 9 [file Table3.docx]

Supplementary Table S4. siRNA sequences.

siRNA sequences used

siMGST1-1:

Sense: GGAUAUGGAGUUACUCUUU

Antisense: AAAGAGUAACUCCAUAUCC

siMGST1-2

Sense: AGUAGAACGUGUACGCAGA

Antisense: UCUGCGUACACGUUCUACU
